# “I Needed for You to See What I’m Talking About”: Experiences With Telehealth Among Homeless-Experienced Older Adults

**DOI:** 10.1177/23337214231172650

**Published:** 2023-05-06

**Authors:** Ali Zahir, Deborah Yip, Cheyenne Garcia, Ashley Nicole Smith, Zena Dhatt, Michael Duke, Margot Kushel

**Affiliations:** 1 University of California, San Francisco, San Francisco, USA; 2 Zuckerberg San Francisco General Hospital and Trauma Center, CA, USA

**Keywords:** homeless, older adults, telehealth, telemedicine, COVID-19

## Abstract

Little is known about how older adults with a current or recent experience of homelessness navigated the switch to telehealth during the COVID-19 pandemic. We examined the perceptions and use of telehealth in a purposive sample of 37 homeless-experienced older adults in mid-late 2020 through semi-structured qualitative interviews. We purposively recruited participants from a larger longitudinal study on homeless-experienced older adults in Oakland, CA. We subjected the data to content analysis. We found that most participants who used telehealth used audio-only phone calls for care. We found that (1) participants experienced challenges accessing the necessary technologies for telehealth, (2) perceptions of telehealth for physical health differed based on the modality (video vs. audio-only), and (3) participants had generally positive perceptions of telehealth for mental healthcare. Our findings suggest that clinicians interacting with homeless-experienced older adults should address the potential skepticism of audio-only telehealth patients, and assess their access to, and knowledge of, video conferencing technology.

## Introduction

During the COVID-19 pandemic, many healthcare settings shifted in-person healthcare appointments to telehealth, defined as real-time telephone or videoconferencing visits between clinicians and patients (Karimi et al., 2022; Khoong et al., 2022). Older age and having a low income are associated with a reduced likelihood of accessing telehealth (Dixit et al., 2022). While most older adults in the United States have access to the necessary equipment for telehealth, such as the internet, a smartphone and a computer, they typically have limited technological ability, which makes using telehealth services difficult (Kruse et al, 2018). While older adults’ limited technological ability can be improved with increased exposure to and training in telehealth technology, this may be resource-intensive. Currently, providing older adults with additional training about telehealth technology or dedicated assistance with such technology is not the standard for telehealth appointments (Kruse et al., 2018; Haimi & Gesser-Edelsburg, 2022).

On a single Homelessness is common among extremely low-income adults and is increasing among older adults (Culhane et al., 2013; Hahn et al., 2006). Over a half of single homeless adults are aged Homelessness is associated with chronic mental and physical health conditions, premature development of geriatric conditions, and high rates of mortality (Brown et al., 2016, 2022; Fazel et al., 2014; Kaplan et al., 2019; Roncarati et al., 2018). Despite the high prevalence of health needs, homeless-experienced (those with current or recent experiences of homelessness) older adults have limited access to non-acute healthcare services (Fazel et al., 2014). They have limited access to computers, mobile phones, and reliable internet connections needed for telehealth, and limited experience in the use of these technologies (Raven et al., 2018).

It is unknown how homeless-experienced older adults navigated the switch to telehealth. Therefore, the purpose of this paper is to examine the perceptions and use of telehealth in a purposive sample of homeless-experienced older adults with access to telephones during the COVID-19 pandemic.

## Methods

### Study Design and Recruitment

We conducted a qualitative sub-study of the HOPE HOME Study, a longitudinal cohort study of homeless-experienced older adults in Oakland, CA (Brown, et al., 2016). The purpose of the sub-study was to assess HOPE HOME participants**’** health and access to healthcare during the COVID-19 pandemic.

To recruit the original HOPE HOME cohort (from which we recruited this study sample), study staff used venue-based sampling to recruit 350 participants (HOPE HOME Wave 1) from all overnight shelters serving homeless adults (*n* = 5); all free and low-cost meal programs serving at least three meals a week (*n* = 5); one recycling center and places where unsheltered homeless people stayed in Oakland, CA in 2013 to 2014. At each location, staff selected participants randomly (Brown, et al., 2016; Burnam & Koegel, 1988, we used the same strategy to recruit an additional 100 participants (HOPE HOME Wave 2) to replace those lost to follow-up or who died. Individuals were eligible for HOPE HOME if they were ≥50 years old (≥53 in Wave 2), homeless at baseline according to the Homeless Emergency Assistance and Rapid Transition to Housing (HEARTH) Act and able to provide informed consent in English, as determined by a teach-back method (Homeless Emergency Assistance and Rapid Transition to Housing Act, 2009; Sudore et al., 2006). Trained study staff conducted in-depth, structured interviews at baseline and every 6 months, with brief monthly check-ins in-between. While all participants were homeless at enrollment, they remained in the parent study regardless of housing status.

For this study, we purposively sampled and recruited 37 HOPE HOME participants who had access to telephones, sampling by current living situation: (1) homeless either in unsheltered settings (outside, vehicles) or congregate shelters (*N* = 12); (2) homeless staying in Shelter-in-Place (SIP) hotel rooms (*N* = 11); or (3) housed (*N* = 14). All potential participants agreed to participate. SIP hotel rooms provided non-congregate shelter to homeless adults who had risk factors for poor outcomes for COVID-19, including being older or having co-occurring health conditions (City and County of San Francisco, 2020). Some SIP hotels provided residents with reliable phone and internet access and healthcare services. Housed participants lived with family/friends or alone. We oversampled women to assess whether their experiences of the COVID lockdown differed from men.

### Data Collection and Analysis

Between July and September 2020, two team members conducted a single 1-hr semi-structured interview with each participant via telephone (see Appendix A for the interview guide). We audio-recorded each interview. We asked participants to find a quiet and secluded space to complete the interview, but in a handful of cases other people (non-participants) were in the same room. A professional transcriptionist transcribed the recordings verbatim. We ceased interviewing when we reached thematic saturation. We provided individuals with a $25 gift card for participating. The University of California, San Francisco’s Institutional Review Board approved all study procedures.

We subjected the data to content analysis to understand its theoretical insights (Although approaches to content analysis vary among qualitative researchers, they all share the perspective that data collection, analysis, and theory generation should be completed simultaneously in an iterative process (Krippendorff, 1980). Consistent with content analysis approaches, we began data analysis concurrently with data collection. We engaged in three interpretative activities: (1) data summarizing and consensus data analysis discussions, (2) codebook development and coding, and (3) data synthesis and manuscript development. First, we created detailed one-page summaries immediately after the completion of each qualitative interview. These summaries included an outline of the participants’ responses as well as theoretical memoing, in which interviewers provide thematic impressions and insights (Phillippi & Lauderdale, 2018). We did not show the memos, interview transcripts, or overall thematic summaries to the participants. The lead qualitative researcher created a preliminary codebook from meetings with the interviewers and review of transcripts.

Three coders independently coded a small subset of interviews and then met together to revise code definitions, delete or collapse codes, and add new codes. Using this iterative process, we revised the codebook and established inter-rater reliability via a schedule in which the research team periodically coded the same transcripts, and subsequently discussed their coding decisions until they reached consensus (O’Connor & Joffe, 2020). We continued to meet regularly to discuss transcript-specific coding questions and possible codebook revisions until we coded all interviews using Dedoose Version 8.0.35 (SocioCultural Research Consultants, LLC, 2018). The final stage of data analysis included consensus discussions with the full analytic and interview team about the salient themes and the presentation of findings. During these analytic discussions, we reviewed each transcript to determine which telehealth modality participants used to access health care (See Table 1 for the list of salient codes and their frequencies). This analysis is descriptive and not intended to propose new theoretical or conceptual constructs.

**Table 1.** Statements About Physical and Mental Health Consultations Via Telephone and Videoconferencing.

Codes	Definition	Consultation type	Attitudinal assessment	Representative quote
Telehealth-Telephone	Experiences with using audio-only telephone health consultations	Physical Health	Positive	No supporting quotes
		Physical Health	Negative	I don’t like having to go to the doctor over the phone, that’s not, he’s not, he can’t see me, he can’t look and see how my leg is doing over no damn telephone and I don’t like that. That don’t even make sense to me, it’s ridiculous if you want to know the truth. (5) I don’t like telehealth appointment, I don’t. You know, it’s impersonal, they’re kind of sterile. I like to see a person’s eyes when I’m having an interview, so far as my health is concerned anyways. . . Because health is a two-way street, it comes from me first and then I have to relay it to the doctor, then the doctor has to make a diagnosis and I can see, you know, whether or not the doctor’s making a real judgment instead of just making some off of the top of your head kind of diagnosis. (9)
		Mental Health	Positive	(A telephone consultation) makes me feel great. It makes me feel good because I open up to her, I talk with her. She’s practically about the only person I really talk to and open up with, is her and my doctor. When I call and I’m depressed and I need to talk, like she said, “M———-, call me and I will call you right back.” (7) I had a therapist that I was speaking to when I was staying at a camp over on 34th and Martin Luther King, a social worker and, but she, you know, she’s basically a friend but she’s a therapist also. And whenever I need someone to talk to I can always call her. (4)
		Mental Health	Negative	No supporting quotes
Telehealth-Videoconferencing	Experiences with using videoconferencing platforms for health consultations	Physical Health	Positive	Participant: Oh, I love (videoconference consultation) because the times that I go actually in the clinic to see them is when they need to do a procedure or something, and I haven’t needed any procedures. (2)
		Physical Health	Negative	(The clinic) tried to set up some kind of a video appointment and I didn’t, I either didn’t know how to make my phone do it or it didn’t come through right so it never happened. (1) I’ve had over the phone appointment. No Zoom, no Zoom because my phone don’t do Zoom. If I have to do some Zoom stuff (my partner) have to show me how to use the computer to get to that. She’s the computer expert in the home. (9)
		Mental Health	Positive	Well, I’ve got a 20 year relationship with my therapist so we kind of know each other. I feel comfortable with her to see my own bullshit, so that’s nice. But, I would say (videoconferencing) it’s a lesser experience, but still it’s been very helpful. (6)
		Mental Health	Negative	No supporting quotes

## Results

The median age was 58 (IQR = 55–60). About half of the participants were men (*n* = 20) and most were Black (*n* = 28). Almost all (*n* = 36) had a chronic health condition; most had two or more (*n* = 34). The median monthly income was $986 (IQR = $636–1,336). (Table 2) Participants expressed that their complex health issues motivated them to attend healthcare appointments during the pandemic. Many participants faced barriers to accessing healthcare during the COVID-19 pandemic. Some common barriers included a lack of transportation to appointments due to decreased public transit schedules and

**Table 2.** Participant Demographics (*n* = 37).

Characteristic	*N* (%) or [IQR]
Median age [IQR]	58 [5]
Gender	
Cis-woman	16 (43.2)
Cis-man	20 (54.1)
Transgender woman	1 (2.7)
Race/ethnicity	
Black	28 (75.7)
White	4 (10.8)
Hispanic/Latino	3 (8.1)
Native American	2 (5.4)
Living situation	
Housed	14 (37.8)
Shelter-in-Place (SIP) Hotel	11 (29.7)
Homeless (not SIP)	12 (32.4)
Vehicle	8 (66.7)
Motel paid for by self	2 (16.7)
On the street	1 (8.3)
Congregate shelter	1 (8.3)
Median income, in USD [IQR]	986 [350]
Physical health	
Self-reported poor health	22 (59.5)
Any chronic health condition	36 (97.3)
2+ chronic health conditions	34 (91.9)
Any ADL impairment^a^	18 (48.7)
Any IADL impairment^b^	8 (21.6)
Mental health	
Any mental health problem^c^	16 (43.2)
Depression	10 (27.0)
Anxiety	10 (27.0)
PTSD	5 (13.5)
Substance use^d^	
Alcohol	3 (8.1)
Amphetamines	11 (29.2)
Cocaine	31 (83.8)
Opioids	11 (29.7)

a Activities of Daily Living (ADL) impairment defined as self-reported trouble completing any of the following tasks: dressing, bathing/showering, eating, getting in/out of bed, using the toilet.

b Instrumental Activities of Daily Living (IADL) impairment defined as self-reported trouble completing any of the following tasks: taking transportation, managing medications, managing money, applying for benefits, setting up a job interview, and finding a lawyer.

c Any mental health problem is defined as endorsement of any of the following in the past 6 months. Depression defined by Center for Epidemiologic Studies Depression Scale (CES-D) score of ≥22. Anxiety defined by ASI score of ≥4. PTSD defined as a score of 4+ on the Primary Care PTSD Screen (PC-PTSD).

d All substance use measures captured moderate-to-high substance use. Alcohol use is defined by the Alcohol Use Disorders Identification Test (AUDIT) score of ≥8. Amphetamine, cocaine, and opioids use is defined by an Alcohol, Smoking, and Substance Involvement Screening Test (ASSIST) score of ≥4.

All participants had the technology (Fewer than half of our participants (*n* = 12) mentioned telehealth specifically. Few participants explicitly reported the type of telehealth they received (audio-only (*n* = 4), videoconferencing (*n* = 4), or both (*n* = 3)). Two additional participants provided information suggesting that they used telehealth services during the COVID-19 pandemic. Among those who mentioned accessing telehealth (we found no differences in the perspectives or experiences of telehealth between those in different living situations.

### Theme 1: Challenges Accessing Appropriate Technologies for Telehealth

Most participants did not have smartphones or computers capable of videoconferencing. Those who had access to smartphones but were unsheltered did not have a stable internet connection needed for videoconference appointments. Thus, most participants who used telehealth relied on audio-only phone calls. The few participants who had access to videoconferencing technologies tended to lack the knowledge or ability to establish a videoconferencing connection with their clinicians. As one unsheltered homeless woman (1) observed, “They [my doctors] tried to set up some kind of a video appointment and I either didn’t know how to make my phone do it or it didn’t come through right, so it never happened.”

### Theme 2: Perspectives on Telehealth for Physical Healthcare Differed by Modality

The small number of participants who used videoconferencing for medical appointments, all of whom were housed, expressed positive sentiments regarding telehealth. They remarked that their videoconferencing telehealth appointments were more convenient than in-person consultations, because they alleviated access barriers for people with limited mobility and helped overcome the challenges of decreased public transportation due to the pandemic: “I love [telehealth visits] because, the times that I go actually in the clinic to see them [clinicians]. . . I only really need to see them when they need to do a procedure or something, and I haven’t needed any procedures.”—2

However, participants who used audio-only phone calls for appointments generally had negative opinions of telehealth. These participants felt that the switch to telehealth compromised the quality of their interactions with clinicians, leaving them skeptical that they could receive an accurate diagnosis. One housed man (3) noted, “I feel if you’re going to talk to a doctor you need to talk to a doctor in-person. . . they need to really be able to. . . see you and give you the right kind of exam that you need, instead of just answering questions on the phone.” The lack of visual communication with audio-only telehealth appointments posed barriers to establishing a relationship with their clinician. A woman residing in a SIP hotel (4) elaborated, saying that, “They [clinicians] don’t look at your blood pressure and your heart rate and your pulse and everything. Now all you do is tell them what’s wrong and they write out a prescription for pills for you. I have never seen anything like this in my life.” The lack of visual cues undermined participants’ full expression of symptoms and feelings. As one housed woman (5) explained, “I needed to be looked at, I needed for you to see what I’m talking about. I don’t need to tell you over no telephone. . .I know that you can’t see pain, but you can see the pain in my face.”

### Theme 3: Mental Health Needs Were Met Through Telehealth

All participants who received mental health services via telehealth had an established relationship with a mental health clinician prior to the COVID-19 pandemic. However, only one homeless participant in either an unsheltered or congregate shelter setting received any mental healthcare. One housed woman (6) said, “I’ve got a 20-year relationship with my therapist, so we kind of know each other. I feel comfortable with her to see my own bull***, so that’s nice.” Participants’ assessments of telehealth for mental health needs were generally positive regardless of whether their sessions occurred through videoconferencing or audio-only phone calls. Many perceived telehealth appointments for mental health to be an adequate substitute for in-person visits, in contrast to their negative views toward telehealth for physical health needs. Participants viewed telehealth as a way to maintain an established connection with their mental health clinician when they could not have an in-person appointment. The housed woman quoted above (6) noted, “I would say that [telehealth therapy] is a lesser experience, but still it’s been very helpful.” One woman living in a SIP hotel (7) said, “She’s [participant’s therapist] practically about the only person I really talk to and open up with. . . when I call and I’m depressed and I need to talk, like she said, ‘M———-, call me and I will call you right back.’ Most doctors say that, they don’t. Mines do.”

Participants with informal mental health support, such as conversations with case managers or support groups, echoed this sentiment, as noted by a housed woman (8): “I have a group that I go to. . .a women’s group, so during the week I have a women’s group in my therapy, and then I have a counselor that checks in with me. So, I do have support.” Telehealth, regardless of modality, allowed participants to maintain established connections with trusted mental health professionals and informal support groups that otherwise would not be possible during the beginning of the pandemic. However, even though all participants had access to a telephone, no one established a new relationship with a mental health clinician during this period. Those who remained homeless (not in SIP hotels) did not have access to mental healthcare, despite the option for telehealth.

## Discussion

In a sample of 37 homeless-experienced older adults who had access to telephones, we found that most participants did not mention accessing telehealth, which implies low uptake of telehealth. Among those who did mention telehealth, most used audio-only calls. Audio-only calls were popular due to participants’ lack of access to phones or computers with videoconferencing capability or their limited knowledge of using videoconferencing applications. Participants who used audio-only telehealth appointments for physical health expressed dissatisfaction, considering them impersonal and poorly suited to providing the visual and interpersonal information necessary for care. Participants who used videoconferencing tended to hold positive views of telehealth, particularly in terms of its convenience relative to in-person appointments. In contrast, participants found telehealth by any modality to be an adequate substitute for appointments with previously known mental health clinicians or support groups. However, telehealth did not appear to overcome barriers to engaging in mental healthcare for those who were not previously engaged, namely those who were staying in unsheltered settings or congregate shelters.

Our participants were extremely low-income older adults with a recent or current experience of homelessness. These individuals experience many barriers to healthcare (e.g., competing needs, transportation, and time) and telehealth may have a role in overcoming some of these barriers (Adams et al., 2021; Davies & Wood, 2018). However, participants did not find audio-only telehealth for physical health encounters to be an adequate substitute for in person visits, despite its ability to surmount transportation barriers. Participants did not trust clinicians’ ability to diagnose and manage physical health conditions via audio only. The negative perceptions may be due to participants’ concern that without visual and tactile information, the clinician could not make a diagnosis, or that audio-only visits do not provide the dyad with emotional cues found during in-person or, possibly, videoconference sessions (Terry & Cain, 2016). In the general population, audio-only telehealth appointments are associated with lower patient understanding and satisfaction than video calls or in-person appointments (Lion et al., 2015; Terry & Cain, 2016; Voils et al., 2018).

Access to and knowledge of how to use videoconferencing hardware and software, Wi-Fi connectivity, and electricity is necessary for videoconferencing telehealth appointments. We found that our participants (all of whom had telephones) experienced barriers to using videoconferencing, including lack of appropriate hardware and technological confidence and knowledge. This finding is similar to other low-income and/or older populations (Anderson & Perrin, 2019; Choi et al., 2022; Perrin & Atske, 2021; Raven et al., 2018; Vogels, 2021). Owning a working computer or tablet, having experience using the internet, and having the ability to learn new technology are associated with increased odds of an older adult adopting telehealth (Choi et al., 2022). Most participants in our study did not have these resources.

Participants endorsed the use of telehealth—both audio-only and videoconferencing—for mental health visits with established clinicians and informal support groups. These results are consistent with studies that find the quality and patient acceptability of telehealth for mental health needs are equivalent to in-person care for many conditions, such as depression and PTSD (Shigekawa et al., 2018). This may be related to participants’ belief that clinicians do not need visual and tactile information when diagnosing and treating mental health conditions, since they rely on guided conversations and verbal assessments. However, we found that no participants established new telehealth-based mental healthcare and only one participant who was homeless in either an unsheltered or congregate shelter setting had a prior relationship with a mental health clinician. This finding may reflect overall barriers to mental healthcare in this marginalized population. We recommend additional outreach and enrollment in mental health services for older adults experiencing homeless.

Our study had several limitations. We restricted our study to participants who had access to telephones. We hypothesize that those without phones would have had no access to telehealth services (Raven et al., 2018). We did not specifically ask about telehealth in our interview guide so it is possible that some participants who did not mention telehealth in their interview did use telehealth services. We interviewed participants at the beginning of the pandemic, when many healthcare settings were not offering in-person visits. Additionally, we did not examine whether homeless-experienced older adults would accept telehealth for new patient visits or prefer it over in-person visits. We cannot exclude that the differences we found in acceptability of videoconferencing was due to different participant characteristics between those who did and did not have access to videoconferencing technology.

Some healthcare providers, such as the U.S. Department of Veterans Affairs, have offered to homeless patients electronic tablets and hands-on training to use the tablets’ videoconferencing features (Garvin et al., 2021). However, these efforts may be of limited utility to individuals who may lack access to the other factors (e.g., access to electricity, internet) necessary to use them. Moreover, some community-based health centers where homeless-experienced older adults receive care may lack the capacity to provide telehealth, regardless of their patients’ technological, infrastructural, and training capabilities (Lin et al., 2018). Older adults with cognitive impairments and impairments in executive function may have a harder time learning these technologies. Because older homeless adults display a higher prevalence of cognitive impairment than the older population as a whole, these differences may also explain the lack of variability in videoconferencing uptake across living situations (Brown et al., 2016).

We found that participants who used videoconferencing perceived that telehealth relieved some barriers to healthcare utilization, such as transportation. However, extremely low-income adults face numerous additional barriers to healthcare use, such as time constraints, caregiving responsibilities, communication barriers, stigma, and medical mistrust (Davies & Wood, 2018; Eaton et al., 2015; Jaiswal & Halkitis, 2019). Increased access to telehealth services and technology does not necessarily translate to a high degree of uptake.^2^ Efforts to increase the use of telehealth to mitigate transportation barriers to healthcare utilization among homeless-experienced older adults and other extremely low-income populations should be done with attention to these barriers.

One way to increase telehealth uptake among homeless-experienced older adults could be for libraries and homeless drop-in centers to offer telehealth stations. These stations could be located in private spaces suitable for healthcare appointments and led by staff who can assist older adults in using videoconferencing technologies. Our findings suggest that clinicians interacting with homeless-experienced older adults could assess for digital literacy barriers and provide tailored education on accessing telehealth services to homeless-experienced older adults, who already face inequities in utilizing healthcare services. Additionally, clinicians should acknowledge and address the potential skepticism of audio-only telehealth patients. As telehealth use expands, policy makers and healthcare organizations need to be intentional about their implementation strategies to ensure that telehealth does not create additional barriers to overcome.

## Appendix A: Relevant Interview Guide Questions

12. Tell me about your physical health. Do you have any health conditions you’re living with?
  Probe: How has your health been since COVID?
  Probe: Has the way you manage your health changed since COVID? How do you feel about these changes?
  Note: avoiding healthcare because of fear of getting COVID in hospitals or other care settings.
  Note: any mention of chronic health conditions; mobility issues; utilization of healthcare, dental care, drug treatment, therapy, treatment, getting medicine, scheduling appointments with PCP or specialty care, visiting the emergency department (room).
13. Let’s talk about your mental health. How have you been feeling emotionally or mentally since COVID?
  Probe: Some people have felt sad or lonely as a result of the pandemic. What do you think about this?
  Probe: Have you tried getting care for your mental health since COVID? If so, tell me about that experience.
  Probe: Are there any positive experiences you’ve had since COVID?
  Probe: What are some things that you do to manage your mental health?
  Note: (e.g., quality of sleep impacted by anxiety, general outlook on the time span of the pandemic (moving timelines of COVID and SIP) impacting mental health, increased social isolation because of SIP; need to physical and social contact; COVID as a stressor resulting in increased anxiety & depression; mistrust of local, state, and national governments)
14. Has COVID changed your ability to get what you need?
  Note: food security– that is, changed to access to food bank; charge your phones, income or work; receiving help from others; receiving medical care; receiving social services [housing navigation, benefits, case management].
  (If yes) How?
15. Is there anything else related to how COVID has changed your life that you’d like to share?
  Notes: Quality of sleep, substance use, getting exercise, socializing.
